# Supplementary material for: The Usability of IT Systems in Document Management, Using the Example of the ADPIECare Dorothea Documentation and Nurse Support System
Source: Int J Environ Res Public Health. 2022 Jul 20;19(14):8805. doi: 10.3390/ijerph19148805 (PMC9323578; doi:10.3390/ijerph19148805)
Supplement: Supplementary file 1 [file ijerph-19-08805-s001.zip › ijerph-1774308-supplementary.pdf]

Supplementary Materials

Evaluation of the usability of the ADPIECare System "Dorothea" – the Survey Questionnaire

1) Enter when the first use of the application took place (one answer possible): *1st year of first-cycle studies (BSc)/ 2nd year of first-cycle studies (BSc)/ 3rd year of first-cycle studies (BSc)/ 1st year of MA studies/ 2nd year of MA studies*

2) The use of the application has taken place both on: (multiple choices): *1st year of first-cycle studies (BSc)/ 2nd year of first-cycle studies (BSc)/ 3rd year of first-cycle studies (BSc)/ 1st year of MA studies/ 2nd year of MA studies*

3) The use of the application was intended to prepare the BSc thesis: (Yes/No)

4) The application was used during the classes: *not once/1 time/2 - 5 times/more than 5 times*

5) The application was used outside the classroom lessons: *not once/1 time/2 - 5 times/more than 5 times*

6) When using the application for the first time, it filled in: (many answers are possible)

7) How long did it take to document the care plan for one patient: *up to 10 minutes/ from 11 - 20 minutes/ from 21 - 60 minutes/ more than 60 minutes*

Additional Sample Usability Post-Test Questions (HIMMSS)

8) The application had clear, clean, uncluttered screen design.	***1***	***2***	***3***	***4***	***5***
	Strongly Agree				***Strongly Disagree***
9) The application kept screen changes to a minimum during completion of a task.	***1***	***2***	***3***	***4***	***5***
10) The application minimised the number of steps it took to complete tasks.	***1***	***2***	***3***	***4***	***5***
11) Information presented on screens was easy to comprehend quickly.	***1***	***2***	***3***	***4***	***5***
12) Information needed for a specific task was grouped together on a single screen.	***1***	***2***	***3***	***4***	***5***
13) Choice lists were clear and unambiguous.	***1***	***2***	***3***	***4***	***5***
14) Clinical documentation tools were efficient to use.	***1***	***2***	***3***	***4***	***5***
15) Alerts were only presented at appropriate times.	***1***	***2***	***3***	***4***	***5***
16) Data could be entered once then used in multiple places.	***1***	***2***	***3***	***4***	***5***
17) I felt confident I could make a mistake without losing my work.	***1***	***2***	***3***	***4***	***5***
	Strongly Agree				***Strongly Disagree***

18) Give three items you found difficult (open-ended question)

19) Provide three items you thought *were easy to use* in the System (open-ended question)

20) The application was used for the first time during the class (please specify the subject) (open-ended question)

21) You would like to use this system for care planning (YES/NO)
